# Immunosuppressive Features of the Microenvironment in Lymph Nodes Granulomas from Tuberculosis and HIV–Co-Infected Patients

**DOI:** 10.1016/j.ajpath.2021.12.013

**Published:** 2022-04

**Authors:** Senait Ashenafi, Jagadeeswara Rao Muvva, Akhirunnesa Mily, Johanna Snäll, Martha Zewdie, Menberework Chanyalew, Anders Rehn, Sayma Rahman, Getachew Aseffa, Amsalu Bekele, Getachew Aderaye, Beede Lema, Mattias Svensson, Susanna Brighenti

**Affiliations:** ∗ Center for Infectious Medicine, Department of Medicine, ANA Futura, Karolinska Institutet, Huddinge, Sweden; † Department of Pathology, Tikur Anbessa Specialized Hospital and Addis Ababa University, College of Health Sciences, Addis Ababa, Ethiopia; ‡ Armauer Hansen Research Institute, Addis Ababa, Ethiopia; § Department of Radiology, Tikur Anbessa Specialized Hospital and Addis Ababa University, College of Health Sciences, Addis Ababa, Ethiopia; ¶ Department of Internal Medicine, Tikur Anbessa Specialized Hospital and Addis Ababa University, College of Health Sciences, Addis Ababa, Ethiopia; ‖ Department of Surgery, Tikur Anbessa Specialized Hospital and Addis Ababa University, College of Health Sciences, Addis Ababa, Ethiopia

## Abstract

Tuberculosis (TB) and HIV co-infection claims many lives every year. This study assessed immune responses in *Mycobacterium tuberculosis*–infected lymph node tissues from HIV-negative and HIV-positive patients compared with the peripheral circulation with a focus on myeloid cells and the cell-signaling enzymes, inducible nitric oxide synthase, and arginase (Arg)-1. Methods included immunohistochemistry or confocal microscopy and computerized image analyses, quantitative real-time PCR, multiplex Luminex, and flow cytometry. These findings indicate enhanced chronic inflammation and immune activation in TB/HIV co-infection but also enhanced immunosuppressive responses. Poorly formed necrotic TB granulomas with a high expression of *M. tuberculosis* antigens were elevated in TB/HIV–co-infected lymph nodes, and inducible nitric oxide synthase and Arg-1 expression was significantly higher in TB/HIV–co-infected compared with HIV-negative TB or control tissues. High Arg-1 expression was found in myeloid cells with a phenotype characteristic of myeloid-derived suppressor cells (MDCS) that were particularly abundant in TB/HIV–co-infected tissues. Accordingly, Lin^−^/HLA-DR^low/int^/CD33^+^/CD11b^+^/CD15^+^ granulocytic myeloid-derived suppressor cells were significantly elevated in blood samples from TB/HIV–co-infected patients. CD15^+^ myeloid-derived suppressor cells correlated with plasma HIV viral load and *M. tuberculosis* antigen load in tissue but were inversely associated with peripheral CD4 T-cells counts. Enhanced chronic inflammation driven by *M. tuberculosis* and HIV co-infection may promote Arg-1–expressing MDSCs at the site of infection thereby advancing TB disease progression.

HIV infection is a major comorbidity of tuberculosis (TB) that is present in 15% of all TB deaths and currently affects approximately 15 million people around the world.^1^ Cellular immunity is gradually destroyed in *Mycobacterium tuberculosis* as well as HIV infection, manifesting in severe depletion of CD4^+^ T cells and dysfunctional activity of macrophages and dendritic cells (DCs). Activation of macrophages and DCs results in elaboration of proinflammatory cytokines, induction of antimicrobial effector pathways, and priming of specific Th1 and cytolytic effector T cells that are required to kill intracellular *M. tuberculosis*. Th1 cytokines, such as IL-1β, tumor necrosis factor-α, and interferon (IFN)-γ, are instrumental in activation, recruitment, and organization of immune cells at the site of *M. tuberculosis* infection, which results in TB granuloma formation. TB granulomas are a hallmark of human TB and defined as dynamic cellular clusters of *M. tuberculosis*–infected macrophages, which are surrounded by a peripheral cuff of T cells and other immune cells.^2^^,^^3^ Although productive granulomas are considered to isolate and limit spread of *M. tuberculosis* infection,^4^ their formation may fail in individuals with a compromised immune system, including TB/HIV–co-infected patients.^5^ This process is likely dependent on the degree of HIV-induced immunosuppression from mild or moderate to more advanced immunosuppression characterized by reduced cell recruitment to the TB granuloma and release of large numbers of *M. tuberculosis* bacilli.^6^

Myeloid cells have a central role in TB and HIV infection, including a highly heterogenous population of mononuclear myeloid cells, such as not only monocytes, macrophages, and DCs but also granulocytic myeloid cells and myeloid-derived suppressor cells (MDSCs).^7^ MDSCs are pathologically activated myeloid cells with immunosuppressive traits that were originally described in the tumor immunology field,^7^ although these cells are also involved in chronic inflammatory conditions, including TB^8^ and HIV infection.^9^ MDSCs comprise a heterogenous population of monocytic or granulocytic subsets that also contain immature progenitors or early-stage MDSCs.^10^ Such myeloid cell subsets impair protective T-cell responses and may contribute to the inability to eradicate these intracellular pathogens at mucosal sites and instead promote persistent infection.^11^

It has been proposed that amino acid metabolism in myeloid cells provides important cues that regulate both innate and adaptive immune responses in different diseases.^12^ Mouse data suggest that classically activated proinflammatory macrophages (M1) produce nitric oxide (NO) and engage in bacterial killing, as opposed to alternatively activated anti-inflammatory macrophages (M2) that instead use arginase (Arg)-1 to induce fibrosis and prohealing responses.^13^^,^^14^ The inducible nitric oxide (iNOS) and Arg-1 regulate L-arginine levels in tissue by competing for cellular L-arginine, which is the substrate for both of these enzymes.^15^ M1 or M2 polarized macrophages stimulate Th1/cellular or Th2/antibody responses, respectively, which further amplify M1/M2 responses via production of IFN-γ or IL-4.^16^ Macrophages and DCs could also express the immunosuppressive enzyme indoleamine 2,3-dioxygenase (IDO) that is involved in tryptophan degradation, which may impair both innate and adaptive immune responses and thus assist microbes to escape eradication by the immune system. Tissue deprivation of L-arginine^17^ or tryptophan^18^ contributes to not only reduced proliferation and activation of T cells but also induction of regulatory T (Treg) cells. iNOS, Arg-1, and IDO are also factors implicated in MDSC-mediated immunosuppression, with the main target being effector T cells.^7^^,^^19^

TB/HIV co-infection often manifests as cervical lymphadenopathy, a finding that is also common in patients infected by TB or HIV alone.^20^ Accordingly, human lymph node tissue and blood from HIV-negative or HIV-positive TB-infected individuals were used to explore the effect of HIV co-infection in TB disease, particularly in TB granuloma formation. Specific subsets of myeloid cells and effector molecules as well as immunosuppressive pathways in the local environment of *M. tuberculosis*–infected lymph nodes were studied.

## Material and Methods

### Study Participants and Diagnosis

Participants were recruited at the Tikur Anbessa University Hospital in Addis Ababa, Ethiopia, after providing signed informed consent. Inclusion criteria were individuals >18 years of age who were HIV negative or HIV positive and had cervical lymph node enlargement and normal chest radiographic findings. Common clinical signs of TB infection, such as fever, sweating, or weight loss, were not used to stratify patients. Exclusion criteria were a history of previous TB or >1 week of antimicrobial chemotherapy, presence of pulmonary involvement consistent with TB, current or prior antiretroviral therapy, or lack of consent to HIV screening. Individuals with nonspecific reactive enlargement of the cervical lymph nodes with no signs of TB disease were recruited as controls. Ethical approval of the study was obtained from the national ethical review boards in Ethiopia and Sweden.

The study participants (*n* = 23) were divided into three groups: i) TB-single infection (*n* = 8), ii) TB/HIV co-infection (*n* = 8) and iii) HIV-negative controls (*n* = 3) and HIV-positive controls (*n* = 4) without TB disease (Table 1). TB diagnosis was based on a combination of histopathological evidence as well as clinical symptoms, including clinical response after completed anti-TB chemotherapy, which was defined as resolution of lymph node enlargement. TB-positive specimens (*n* = 16) revealed a granulomatous inflammation with epithelioid cell clusters, multinucleated giant cells, and caseating necrosis consistent with TB. The histologic features of TB-negative control lymph node samples (*n* = 7) typically demonstrated a reactive lymphoid hyperplasia with no granuloma formation.

**Table 1:** Clinical and Laboratory Characteristics of Study Participants

Characteristic∗	TB/HIV-negative patients (*n* = 8)	TB/HIV-positive patients (*n* = 8)	*P* value†	Controls (*n* = 7; 4 HIV positive)	*P* value‡	*P* value§
Age, years	30 (20–46)	34 (25–49)	>0.999	28 (20–40)	>0.999	0.423
Male/female sex	5/3	5/3		3/3 (1 NA)		
Histology, *n* (%)¶	8 (100)	8 (100)		0 (0)		
TST, mm	17 (13–25)	17 (0–25)		10 (0–20)		
QFT, IU	11 (0.32–23.4)	3.3 (0.34–7.9)	0.152	ND		
CD4 T-cell counts, cells/mm^3^	677 (448–1277)	203 (52–488)	0.001	457 (230–886)	0.121	0.021
CD8 T-cell counts, cells/mm^3^	419 (168–670)	818 (368–1528)	0.072	1048 (389–1961)	0.007	0.367
CD4:CD8 ratio	1.61 (0.68–2.67)	0.24 (0.10–0.34)	0.0003	0.53 (0.13–1.13)	0.002	0.114
Viral load, copies/mL	ND	254,000 (20,000–550,000)		30,500 (12,000–72,000)		0.024
Hemoglobin, g/dL	15 (12.4–21.4)	11 (8.0–13.7)	0.021	14 (10.6–16.7)	0.977	0.046
WBC count, mm^3^	5851 (4100–7100)	9400 (3800–13,300)	0.050	6450 (4300–8900)	0.628	0.132
ESR, mm/h	28 (5–50)	81 (30–130)	0.011	22 (1–56)	0.609	0.008

ESR, erythrocyte sedimentation rate; NA, not available; ND, not determined; QFT, QuantiFERON-TB Gold in-Tube; TB, tuberculosis; TST, tuberculin skin test; WBC, white blood cell.

∗ Data are presented as mean (range) unless otherwise stated.

† TB versus TB/HIV (Kruskal-Wallis test and Dunn posttest).

‡ TB versus controls (Kruskal-Wallis test and Dunn posttest).

§ TB/HIV versus controls (Kruskal-Wallis test and Dunn posttest).

¶ Histologically confirmed TB diagnosis of excisional biopsy of cervical lymph nodes showing typical morphological changes consistent with TB.

### Clinical Samples and Procedures

An excisional biopsy of a single enlarged cervical lymph node was obtained from each study participant, along with matched peripheral blood. Lymph node biopsy specimens were divided into two parts for future analysis: tissue fixed in formalin (Sigma-Aldrich, St. Louis, MO) and embedded in paraffin blocks and tissue preserved in RNAlater (BioRad, Hercules, CA) at −85°C. Hematoxylin and eosin (H&E) (Sigma-Aldrich)–stained tissue sections were used for histopathological diagnosis of the lymph node samples. Plasma samples were used for HIV screening and determination of plasma HIV viral load, according to the national guidelines in Ethiopia. Blood chemistry analyses included assessment of hemoglobin, white blood cell counts , and erythrocyte sedimentation rate (ESR). Immunologic status was assessed using the tuberculin skin test (Statens Serum Institut, København, Denmark), whereas whole blood samples were used for peripheral CD4/CD8 T-cell counts (FACSCount; BD Biosciences, Franklin Lakes, NJ), and IFN-γ production was measured with the QuantiFERON-TB Gold In-Tube (Cellestis, Victoria, Australia) according to the manufacturer's instructions. A tuberculin skin test reaction measured at 48 to 72 hours after tuberculin (Statens Serum Institute) injection was considered a positive result when the transverse induration was ≥10 mm for TB and ≥5 mm for TB/HIV–co-infected patients, whereas QuantiFERON-TB Gold In-Tube results above a cut-off of 0.35 IU/mL were considered positive.

### Antigen Retrieval in Paraffin-Embedded Lymph Node Tissues

Formalin-fixed, paraffin-embedded tissue biopsy specimens were cut into 3-μm-thick sections and mounted on Superfrost Plus microscope slides (Thermo Fisher Scientific/Menzel-Gläser, Waltham, MA). The tissue was deparaffinised using xylene (Thermo Fisher Scientific) for 3 × 5 minutes followed by rehydration with decreasing concentrations of ethanol (Solveco, Stockholm, Sweden) at 100% for 2 × 5 minutes, 95% for 5 minutes, and 70% for 5 minutes. Dewaxed slides were rinsed with Tris-buffered saline (TBS; Sigma-Aldrich), and endogenous peroxidase was blocked with 3% H_2_O_2_ (Sigma) for 30 minutes before antigen retrieval was performed by microwave boiling in 0.01 mol/L citrate buffer (pH 6) (Sigma-Aldrich) for 2 × 2.5 minutes. After cooling and washing in TBS, sectioned tissues were used for immunostains as described below.

### Immunostaining of Lymph Node Tissues

Deparaffinized and rehydrated tissue sections were blocked for 30 minutes with 5% fetal calf serum (Sigma-Aldrich) in TBS before staining with immunohistochemistry (IHC) or immunofluorescence (IF). IHC was performed using the Vectastain Elite ABC-kit (Vector Laboratories, Burlingame, CA) as previously described,^21^, ^22^, ^23^ and positive stain was developed using a diaminobenzidine substrate (Vector Laboratories) and hematoxylin (Sigma-Aldrich) for nuclear counterstaining. For co-localization of immune molecules, 2- to 4-color stains were performed using indirect IF and DAPI mounting media (Vector Laboratories) for visualization of cell nuclei.

Primary antibodies used for IHC were monoclonal mouse anti-human CD3, CD4, and CD8 (BD Biosciences, San Jose, CA), CD20 and collagen type I (Abcam, Cambridge, UK), CD68 and CD163 (Novocastra, Leica Biosystems, Wetzlar, Germany), monoclonal rabbit anti-human CD11b and CD11c (Abcam) and polyclonal rabbit anti-human iNOS (Thermo Fisher Scientific), Arg-1 and CD163 (GeneTex, Irvine, CA), and IDO-1 (Sigma). A polyclonal rabbit anti–*M. tuberculosis* bacteria antibody (Invitrogen, Waltham, MA) was used for detection of *M. tuberculosis*, whereas a polyclonal rabbit anti-HIV-1 p55+p24+p17 antibody (BIOSS Antibodies, Boston, MA) was used for detection of HIV antigens in tissue. Biotinylated secondary antibodies for IHC included goat anti-mouse IgG and swine anti-rabbit F(ab^’^)_2_ from Dako Cytomation (Jena, Germany). Tissue sections stained with secondary antibodies only were used as negative controls. Primary antibodies used for IF were monoclonal mouse anti-human CD68, CD163, CD33, CD3, CD56, CD123 (Novocastra), CD15, CD20, MAC387 (Dako), HLA-DR (Abcam), polyclonal rabbit anti-human iNOS (Thermo Fisher Scientific), Arg-1, and CD163 (GeneTex). Primary antibodies were added and incubated for 90 minutes (polyclonal antibodies) or overnight (monoclonal antibodies) in room temperature. Before addition of the secondary antibodies, tissue sections were washed with TBS and blocked for 30 minutes with 1% normal goat serum (Sigma-Aldrich) in TBS. The secondary antibodies were added and incubated for 30 minutes (IF) or 1 hour (IHC) in the dark. Fluorophore-conjugated secondary antibodies used for IF included goat anti-mouse IgG Alexa-488, goat anti-mouse IgG2b Alexa/Fluor-555, and goat anti-mouse IgG1 Alexa/Fluor-647 (Invitrogen), goat pAb to Ms IgM Daylight-488 (Abcam), as well as goat anti-rabbit IgG Alexa-594 (Molecular Probes, Eugene, OR).

### Quantitative Image Analysis of Lymph Node Tissues

Acquired computerized image analysis was used to quantify IHC staining by transferring digital images of the stained tissue samples from a DMR-X microscope to a computerized Quantimet 5501W image analyzer (Leica Microsystems). Positive immunostaining was quantified at the single-cell level in 10 to 50 high-power fields with a mean total area of 1.5 × 10^5^ μm/field, using the Qwin 550 v.2 software program (Leica Imaging Systems, Wetzlar, Germany). Approximately 50 microscopy fields were assessed in TB- and TB/HIV-infected lymph nodes and approximately 10 to 30 fields for control lymph nodes, which were mostly smaller compared with the TB-infected lymph nodes. Protein expression was determined as the percentage positive area of the total relevant cell area defined as the nucleated and cytoplasmic area within the tissue biopsy, excluding fibrotic and necrotic tissue areas. Occasionally, positive immunostaining determined inside the TB granulomas was compared with tissue outside the granuloma using a tissue excluder function of the software.

### Confocal Analysis of Lymph Node Tissues

For quantification of IF stains, a Nikon A1R confocal microscope coupled to a 32-channel spectral detector was used (Nikon Instruments, Tokyo, Japan). Reference spectra were collected for each fluorophore by imaging samples single stained with the respective probes, using a single excitation source. The reference spectra were stored in a spectral library and used for off-line unmixing of all samples imaged by the spectral unmixing algorithm in the NIS elements AR software version 4.50.00 (Nikon Instruments). For analysis of colocalization, the freeware ImageJ version 2.0.0 (NIH, Bethesda, MD; *http://imagej.nih.gov/ij*) was used. In short, each unmixed channel was thresholded and segmented. Next, the segmented area was measured and masked before the masked areas were transformed into 8-bit images and colocalization assessed using the colocalization plugin, which highlights colocalized points of two 8-bit images. The area of the colocalized points was measured and, in the case of triple-colocalization, masked and transformed into a new 8-bit image. The new image was subsequently analyzed for colocalization against a third channel, using the same plugin.

### Light Microscopy Analysis of H&E-Stained Lymph Node Tissues

H&E stain and light microscopy were used to assess the different types of granulomas in *M. tuberculosis*–infected lymph node tissues (ie, *n* = 8 TB-infected and *n* = 8 TB/HIV–co-infected lymph nodes). Specific granulomas in the lymph nodes were counted and visually graded using developmental stages 1 to 4 (Table 2). Accordingly, a total of 164 granulomas in TB-infected and 117 granulomas in TB/HIV lymph node biopsy specimens were manually counted and graded using a modified version of a previously described histopathological staging of mycobacterial granulomas.^24^

**Table 2:** Histopathological Staging of Granulomas in Lymph Node Tuberculosis

Granuloma	Appearance	Necrosis
Stage 1	Irregular clusters of epithelioid cells interspersed with lymphocytes	Absent
Stage 2	Epithelioid cell aggregates with well-defined borders	Absent or minimal necrotic areas
Stage 3	Epithelioid cell aggregates with well-defined borders	Limited areas of central caseous necrosis
Stage 4	Multicentric epithelioid cell clusters with irregular borders	Extensive areas of caseous necrosis

Lymph node granulomas were examined and graded using a modified version of a previously described histopathologic staging of mycobacterial granulomas in hematoxylin and eosin–stained tissue sections.^24^

### BCG-Specific IgG Enzyme-Linked Immunosorbent Assay

The release of mycobacteria-specific IgG antibodies in plasma samples or *in vitro* peripheral blood mononuclear cell (PBMC) cultures obtained from the study participants was quantified using a BCG-specific enzyme-linked immunosorbent assay. Briefly, a BCG vaccine antigen (Japan BCG Laboratories, Tokyo, Japan) was used to coat Maxisorb plates (Nunc, Roskilde, Denmark) overnight at 4°C. The plates were washed with 0.05% Tween-20 (Sigma-Aldrich) and blocked with 10% fetal calf serum in phosphate-buffered saline. Diluted plasma samples or PBMC culture supernatants (from PBMCs cultured without exogenous stimulation for 48 hours) from patients and controls were added (100 μL per well) and incubated for 2 hours at 37°C before washing and adding a rabbit anti-human IgG horseradish peroxidase conjugate (Jackson Immunoresearch Laboratories, West Grove, PA) for 2 hours at room temperature. Dilution buffer was used as a negative control. The enzyme-substrate reaction was developed after 20 minutes using an O-phenylenediamine (Sigma-Aldrich) substrate solution. BCG-specific IgG titers were expressed as 492 OD and multiplied by the dilution factor.

### Multiplex Luminex Assay

Inflammatory mediators [IL-1 receptor antagonist (IL-1RA), CXCL9, and CXCL10] in plasma samples were assessed using a human standard cytokine 25-plex panel (LHC0009) from Invitrogen according to the manufacturer's instructions using the Bio-Plex Luminex detection system and the Bio-Plex manager 4.0 curve fitting software (Bio-Rad Laboratories, Hercules, CA).

### mRNA Extraction and Quantitative Real-Time PCR

PBMCs or lymph node tissue were saved in RNAlater until mRNA was extracted using the Ambion RiboPure extraction kit (Life Technologies, Carlsbad, CA) according to the manufacturer's instructions. RNA was reverse transcribed to cDNA using superscript reverse transcriptase (SuperScript VILO cDNA master mix, Invitrogen). Amplification of target genes in the cDNA samples was performed using the ABI PRISM 7700 Sequence Detection System, including the 7500 software version 2.0.6 (Applied Biosystems, Foster City, CA). Commercial FAM dye–labeled Taq-Man MGB probes and primers for *Tim-3*, *Tbet*, and *GATA3* were from Sigma-Aldrich, whereas the other primers and probes were from Applied Biosystems. Cycle threshold values for the target genes were normalized to the C_T_ value for the housekeeping gene 18S, and the results were analyzed by using the relative standard method as previously described.^22^^,^^25^ Data were presented as mRNA fold change in TB-infected or TB/HIV–co-infected samples compared with uninfected control samples (*n* = 5, matched PBMC and lymph nodes tissue samples).

### Flow Cytometry

Frozen PBMC samples were thawed and washed with FACS buffer [PBS containing 0.5% (v/v) fetal calf serum and 0.5 mmol/L EDTA] before cells were counted and stained. A total of 1 × 10^6^ cells were stained for 30 minutes at 4°C with fluorochrome-conjugated anti-human antibodies: CD33 (phosphatidylethanolamine), CD11b (phosphatidylethanolamine-Cy7), HLA-DR (phosphatidylethanolamine-Cy5), CD15 (fluorescein isothiocyanate), CD14 (Brilliant Violet 711), and the death cell marker (Zombie UV), whereas CD3, CD19, and CD56 were assessed together in the DUMP channel (Brilliant Violet 510). CD33 and Zombie UV were obtained from Biolegend (San Diego, CA), whereas the other antibodies were from BD Pharmingen (San Diego, CA). Unstained cell samples as well as fluorescence minus one controls were used to determine the background fluorescence and to set the appropriate gates. Cells were washed twice with FACS buffer after staining and fixed with 4% formaldehyde (Sigma-Aldrich) at room temperature for 10 minutes. After fixation, cells were washed twice with FACS buffer, and 50,000 cells were acquired from each sample by using BD LSR Fortessa (BD Biosciences) and analyzed with FlowJo version 9 (BD, Ashland, OR).

### Statistical Analysis

Data are presented as medians ± interquartile ranges or medians ± ranges. Nonparametric analyses were used to calculate indicated *P* values and included a Kruskal-Wallis test and Dunn posttest (when comparing more than two groups) or a Mann-Whitney *U*-test (when comparing two unmatched groups) or a Wilcoxon signed-rank test (when comparing two matched groups). The Spearman correlation test was used for the correlation analyses. A value of *r* = 1 indicates a perfect positive correlation, whereas *r* = −1 indicates a perfect negative correlation. Statistical analyses were performed in GraphPad Prism software version 5 (GraphPad Software, San Diego, CA).

## Results

### Enhanced Systemic Inflammation and Elevated Proportion of Poorly Formed and Necrotic Granulomas in Lymph Node Tissue from TB/HIV–Co-Infected Patients

Clinical and laboratory features of HIV-negative and HIV-positive patients with lymph node TB and controls are summarized in Table 1. As expected, TB/HIV–co-infected patients had significantly lower CD4^+^ T-cell counts (*P* = 0.001) and relatively higher CD8^+^ T-cell counts in peripheral blood, resulting in a lower CD4:CD8 ratio (*P* = 0.0003) compared with HIV-negative patients with TB. Furthermore, hemoglobin levels were lower (*P* = 0.02) and white blood cell counts (*P* = 0.05) and erythrocyte sedimentation rates (*P* = 0.01) were higher in HIV-positive compared with HIV-negative patients with TB. Plasma HIV viral load was higher in TB/HIV–co-infected compared with HIV-positive controls (*P* = 0.02), which may suggest that viral replication is less controlled in TB/HIV co-infection.

**Figure 1:**
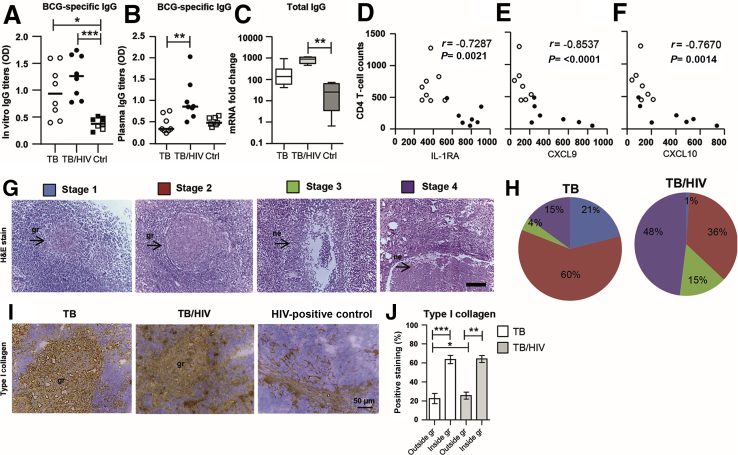
Characterization of systemic inflammation and tissue pathology in tuberculosis (TB)–infected and TB/HIV–co-infected patients. **A–C:** BCG-specific and total IgG were assessed using enzyme-linked immunosorbent assay of peripheral blood mononuclear cell (PBMC) culture supernatants (**A**) and serum samples (**B**), respectively, as well as real-time PCR of freshly isolated PBMCs (**C**) from TB-infected patients, TB/HIV–co-infected patients, and controls (Ctrls). **D–F:** Peripheral CD4 T-cell counts were inversely correlated to inflammatory mediators in plasma, including IL-1 receptor antagonist (IL-1RA) (**D**), CXCL9 (**E**), and CXCL10 (**F**) as assessed using the Spearman correlation test. **G** and **H**: Hematoxylin and eosin (H&E) stain of lymph node tissue sections from a TB/HIV–co-infected patient where the **arrows** indicate solid or necrotic TB granulomas (**G**) that were manually counted and graded as stage 1 to 4 granulomas in TB-infected and TB/HIV–co-infected tissues (**H**). **I** and **J**: Type I collagen staining in TB-infected, TB/HIV–co-infected, and HIV-positive Ctrl tissue (**I**) was used to determine the level of fibrosis in TB- and TB/HIV–co-infected lymph nodes (**J**). **G** and **I**: Representative images of H&E as well as type I collagen staining are shown. Data are expressed as medians ± ranges or interquartile ranges. *n* = 8 TB-infected patients (open circles and white bars); *n* = 8 TB/HIV–co-infected patients (closed circles and light gray bars); *n* = 3 HIV-negative Ctrls (open squares); *n* = 4 HIV-positive Ctrls (closed squares). ∗*P* < 0.05, ∗∗*P* < 0.005, and ∗∗∗*P* < 0.001 (Kruskal-Wallis and Dunn multiple comparisons tests or Mann-Whitney *U*-test or Wilcoxon signed-rank test). Scale bar = 50 μm. Original magnification, ×25. gr, granuloma; ne, necrosis.

**Supplemental Figure S1:**
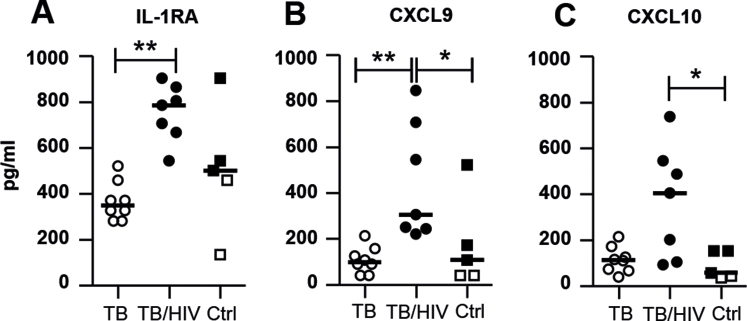
Assessment of inflammatory mediators in plasma, including IL-1 receptor antagonist (IL-1RA) (**A**), CXCL9 (**B**), and CXCL10 (**C**) using multiplex Luminex. Data are expressed as medians ± ranges. *n* = 8 tuberculosis (TB)–infected patients (open circles); *n* = 8 TB/HIV–co-infected patients (closed circles); *n* = 3 HIV-negative controls (open squares); *n* = 4 HIV-positive controls (closed squares). ∗*P* < 0.05, ∗∗*P* < 0.005 (Kruskal-Wallis and Dunn multiple comparisons tests or Mann-Whitney *U*-test).

Elevated mycobacteria-specific IgG production from PBMCs cultured *in vitro* was observed in both TB-infected (*P* = 0.02) and TB/HIV–co-infected (*P* = 0.0006) patients compared with the controls (Figure 1A). TB/HIV–co-infected patients also had enhanced mycobacteria-specific IgG responses in plasma (*P* = 0.0031) as well as elevated total IgG mRNA in lymph node tissue (*P* = 0.002) compared with HIV-negative patients with TB or controls, respectively. Multiplex profiling of plasma samples revealed an altered systemic cytokine/chemokine milieu in TB/HIV–co-infected patients, characterized by significantly elevated levels of the anti-inflammatory IL-1RA (*P* = 0.0038), and increased levels of the IFN-γ–stimulated chemokines CXCL9 (*P* = 0.004) and CXCL10 (*P* = 0.041) compared with HIV-negative patients with TB as well as the controls (Supplemental Figure S1, A–C). Accordingly, there was a significant inverse correlation between CD4^+^ T-cell counts and IL-1RA (*P* = 0.002), CXCL9 (*P* < 0.0001), and CXCL10 (*P* = 0.0014) (Figure 1, D–F), supportive of dysregulated immune activation in TB/HIV–co-infected patients.

Next, microscopy and computerized image analysis were applied to assess the morphology of a total of 281 TB granulomas (164 TB infected and 117 TB/HIV co-infected) in *M. tuberculosis*–infected lymph node tissues. Four major types of granulomas were observed and presented as stages 1 to 4, with and without necrosis (Table 2 and Figure 1G). Overall, 81% of the TB granulomas in HIV-negative lymph nodes had well-defined borders and little necrosis (stage 1 to 2), whereas disrupted cellular aggregates with extensive necrosis (stage 3 to 4) were more common in TB/HIV co-infection and observed in 63% of the granulomas (Figure 1H). Quantification of tissue fibrosis using type I collagen showed that both HIV-negative TB-infected and TB/HIV–co-infected lymph nodes contained enhanced fibrosis compared with control tissues (Figure 1I). Fibrosis was particularly high in the granulomatous areas (Figure 1I), and image analyses confirmed that granulomas in both groups demonstrated significantly elevated levels of type I collagen (*P* = 0.0006 and *P* = 0.008, respectively) (Figure 1J) compared with the surrounding tissue. Type I collagen expression in tissue outside the granulomas was also significantly higher (*P* = 0.02) in TB/HIV co-infection compared with TB infection (Figure 1I). Altogether, these results suggested that persistent immune activation and chronic inflammation, including poorly formed and necrotic lymph node granulomas, were more common in TB/HIV–co-infected patients compared with HIV-negative patients with TB.

### Elevated Expression of *M. tuberculosis* Antigen in *M. tuberculosis*–Infected Tissues

**Figure 2:**
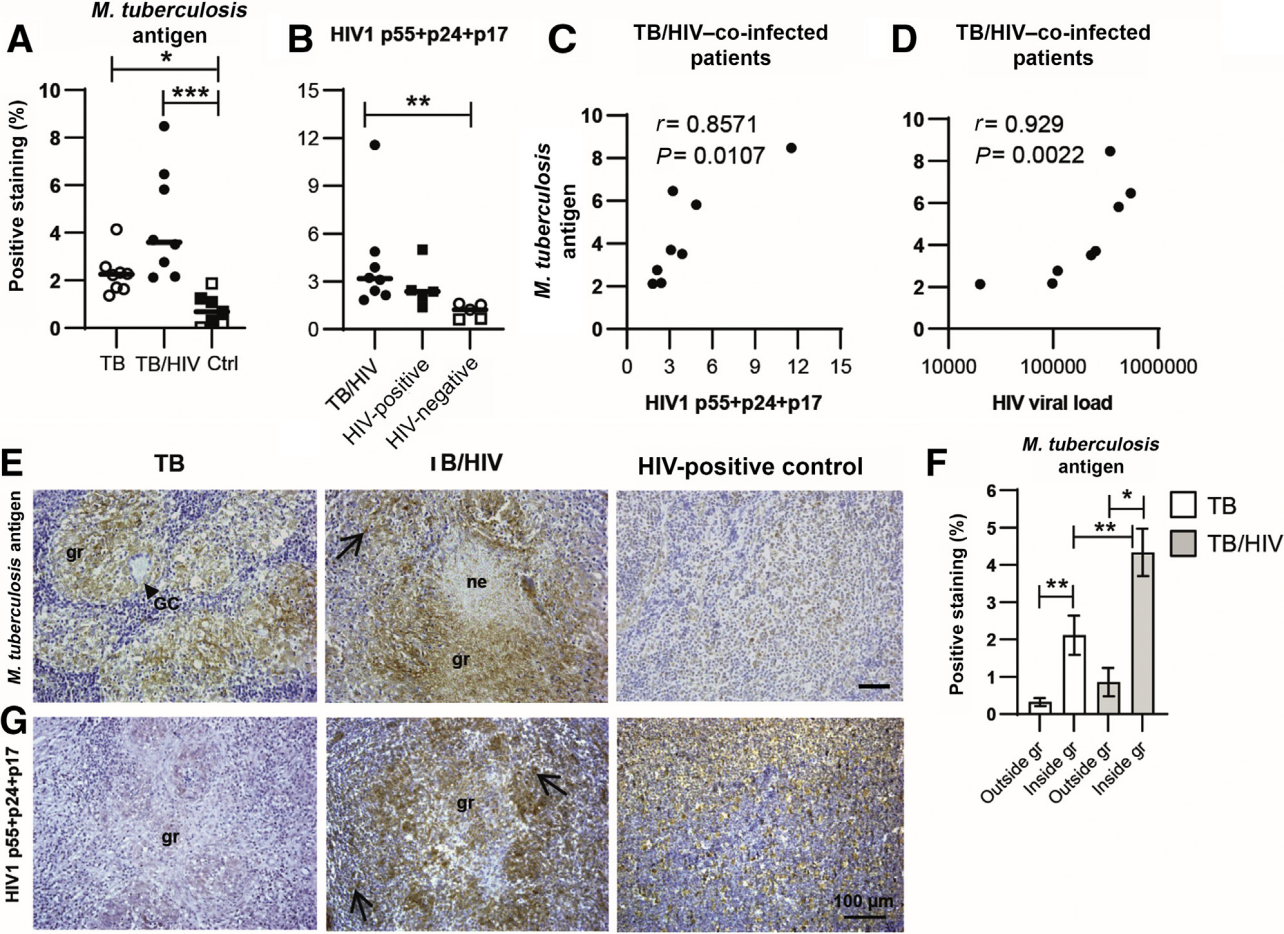
*In situ* quantification of *Mycobacterium tuberculosis* and HIV1 antigen load in tuberculosis (TB)–infected and TB/HIV–co-infected lymph node tissues. **A** and **B**: Immunohistochemistry and computerized image analyses was used to determine the expression (percentage of positive staining in the total cell area) and distribution of *M. tuberculosis* antigen (**A**) and HIV1 p55+p24+p17 (**B**). Note that HIV antigen stain in TB/HIV co-infection is compared with HIV-positive and HIV-negative controls (Ctrls) (3 TB-infected patients and 2 HIV-negative Ctrls). **C** and **D:***M. tuberculosis* antigen expression in TB/HIV–co-infected tissue correlated to HIV antigen load in tissue (**C**) and to HIV viral load in plasma (**D**) as assessed using the Spearman correlation test. **E** and **F**: Representative images of M. tuberculosis–positive staining in TB-infected, TB/HIV–co-infected, and HIV-positive Ctrl tissue (**E**) and image analyses of M. tuberculosis antigen expression outside and inside the granulomatous areas in TB-infected and TB/HIV–co-infected patients is shown (**F**). **G:** Representative images of HIV1 p55+p24+p17–positive staining in TB-infected, TB/HIV–co-infected, and HIV-positive Ctrl tissue. **E** and **G: Arrows** in the images indicate positively stained cells, and **arrowheads** indicate giant cells (GCs). Data are expressed as medians ± ranges or interquartile ranges. *n* = 8 TB-infected patients (open circles and white bars); *n* = 8 TB/HIV–co-infected patients (closed circles and light gray bars) (**A**, **B** and **F**); *n* = 3 HIV-negative Ctrls (open squares); *n* = 4 HIV-positive Ctrls (closed squares). ∗*P* < 0.05, ∗∗*P* < 0.005, and ∗∗∗*P* < 0.001 (Kruskal-Wallis and Dunn multiple comparisons tests or Mann-Whitney *U*-test or Wilcoxon signed-rank test). Scale bar = 100 μm. Original magnification, ×16. gr, granuloma; ne, necrosis.

To quantify local bacterial burden as well as viral load in the study participants, *M. tuberculosis* and HIV-1 antigen expression was assessed in the lymph node tissues. *M. tuberculosis* antigen was significantly higher in both TB-infected (*P* = 0.05) and TB/HIV–co-infected (*P* = 0.0004) tissues compared with the controls, whereas *M. tuberculosis* antigen load was relatively higher in TB/HIV–co-infected compared with HIV-negative TB-infected tissues (Figure 2A). *In situ* quantification of the HIV-1 antigens p55+p24+p17 showed a significantly (*P* = 0.006) higher expression in TB/HIV–co-infected compared with HIV-negative tissues (Figure 2B). In TB/HIV–co-infected patients, *M. tuberculosis* antigen expression in tissue correlated with HIV-1 antigen load in tissue (*r* = 0.86, *P* = 0.0107) as well as HIV viral load in plasma (*r* = 0.93, *P* = 0.0022) (Figure 2, C and D). Microscopy and image analysis revealed that *M. tuberculosis* antigen expression in both TB-infected and TB/HIV–co-infected lymph nodes was significantly higher in the granulomatous areas compared with the surrounding tissue (*P* = 0.0078 and *P* = 0.016, respectively) (Figure 2E and F). *M. tuberculosis* antigen was also significantly higher in TB/HIV–co-infected granulomas compared with HIV-negative TB granulomas (*P* = 0.0095) (Figure 2F). Tissue expression of HIV-1 antigens was detected in TB/HIV granulomas but also in the lymphoid areas of both TB/HIV–co-infected and HIV-positive study participants (Figure 2G). These results support the notion that HIV can fuel *M. tuberculosis* replication and antigen deposition in co-infected macrophages at the site of infection.^6^

### Altered Cellular Composition in TB/HIV–Co-Infected Lymph Nodes

**Figure 3:**
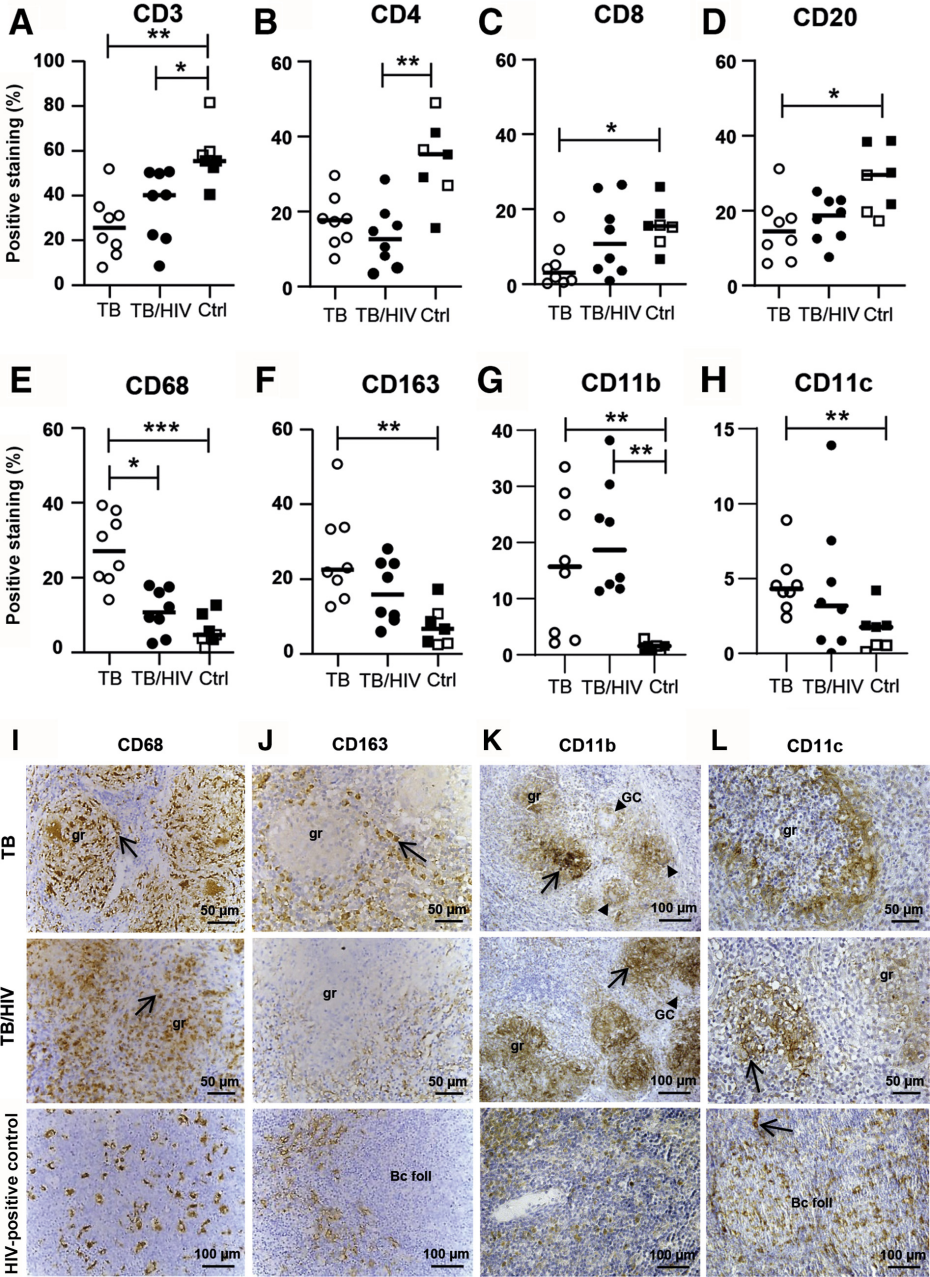
Immune cell composition and morphology in tuberculosis (TB)-infected and TB/HIV–co-infected lymph node tissues. **A–H:** Immunohistochemistry and computerized image analyses was used to determine the expression (percentage of positive staining in the total cell area) and distribution of lymphocytes (**A**–**D**) and myeloid cells (**E**–**H**): CD3 (**A**), CD4 (**B**), and CD8 T cells (**C**), CD20 B cells (**D**), as well as CD68 (**E**), CD163 (**F**), CD11b (**G**), and CD11c (**H**) myeloid subsets. **I–L:** Representative images of CD68-positive (**I**), CD163-positive (**J**), CD11b-positive (**K**), and CD11c-positive (**L**) staining in TB-infected, TB/HIV–co-infected, and HIV-positive control (Ctrl) tissue. **Arrows** in the images indicate positively stained cells, and **arrowheads** indicate giant cells (GCs). Data are expressed as medians ± ranges; *n* = 8 TB-infected patients (open circles); *n* = 8 TB/HIV–co-infected patients (closed circles); *n* = 3 HIV-negative Ctrls (open squares); *n* = 4 HIV-positive Ctrls (closed squares). ∗*P* < 0.05, ∗∗*P* < 0.005, and ∗∗∗*P* < 0.001 (Kruskal-Wallis and Dunn multiple comparisons tests). Original magnification: ×16 (**I, top and middle rows**; **J, top and middle rows; L, top and middle rows**); ×25. Scale bars, 50 μm (**I, top and middle rows**; **J, top and middle rows; L, top and middle rows**); 100 μm (**I, bottom row; J, bottom row; K; L, bottom row**). Bc foll, B-cell follicle; gr, granuloma.

**Supplemental Figure S2:**
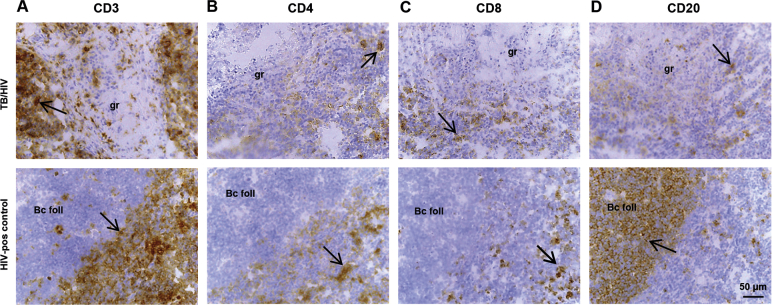
Immunohistochemistry was used to determine the expression and distribution of CD3 (**A**), CD4 (**B**), D8 T cells (**C**), and CD20 B cells (**D**) in tuberculosis (TB)/HIV–co-infected and HIV-positive control tissue. Representative images of CD3, CD4, CD8, and CD20 immunostainings in TB/HIV–co-infected and HIV-positive control tissues are shown. **Arrows** indicate positively stained cells. Scale bar = 50 μm. Original magnification, ×25. Bc foll, B-cell follicle; gr, granuloma.

IHC (Supplemental Figure S2, A–D) and image analyses revealed a significant reduction (*P* = 0.002) of CD3^+^ T cells in both TB-infected and TB/HIV–co-infected lymph nodes (Figure 3A). Although CD4^+^ T cells were significantly reduced in TB/HIV co-infection (*P* = 0.007) (Figure 3B), CD8^+^ T cells were significantly reduced in HIV-negative TB tissues (*P* = 0.04) (Figure 3C). TB-infected (*P* = 0.03) and TB/HIV–co-infected patients had reduced levels of CD20^+^ B cells in lymph node tissue compared with the controls (Figure 3D), and these reduced levels were particularly pronounced in HIV-negative TB-infected tissues.

*In situ* expression and distribution of different myeloid markers showed a significant increase of CD68^+^ (*P* = 0.0007) and CD163^+^ (*P* = 0.005) macrophages as well as CD11b^+^ (*P* = 0.0097) and CD11c^+^ (*P* = 0.031) cells in HIV-negative TB-infected lymph nodes tissues compared with controls (Figure 3, E–H). CD68^+^ macrophages were also higher in TB-infected compared with TB/HIV–co-infected tissues (*P* = 0.001) (Figure 3E). The only myeloid marker that was up-regulated in TB/HIV–co-infected tissues compared with the control was CD11b (*P* = 0.0022), whereas CD68, CD163, and CD11c were all relatively lower compared with HIV-negative TB tissues (Figure 3, E–H). CD68^+^ granulomas with well-defined borders were less frequent in TB/HIV–co-infected lymph nodes (Figure 3I) and CD68^+^ cells were confined inside the *M. tuberculosis* granulomas, whereas CD163^+^ cells were located at the periphery, surrounding CD68^+^ macrophage clusters (Figure 3, I and J). Most CD11b^+^ and CD11c^+^ cells were located in the granulomatous lesions of TB-infected and TB/HIV–co-infected tissues, but some were also distributed in the lymphoid areas of the lymph nodes (Figure 3, K and L). The size and morphology of CD11b^+^ cells were usually smaller and more rounded compared with CD11c^+^ cells, which were larger and more DC-like in the patient as well as control tissues (Figure 3, K and L). Overall, altered cellular dynamics in TB-infected and TB/HIV–co-infected lymph nodes involved extensive tissue remodeling, including reduced expression of both CD4^+^ and CD8^+^ T cells and CD20^+^ B cells but elevated myeloid cell subsets. A lower expression of CD68 and the M2-associated CD163 receptor but elevated levels of the cell surface integrin CD11b in TB/HIV–co-infected tissues may suggest the presence of an aberrant myeloid cell population in these patients.

### mRNA Expression Profiling Suggests Elevated Inflammation and Increased Immune Inhibition at the Site of Infection in TB/HIV–Co-Infected Patients

**Figure 4:**
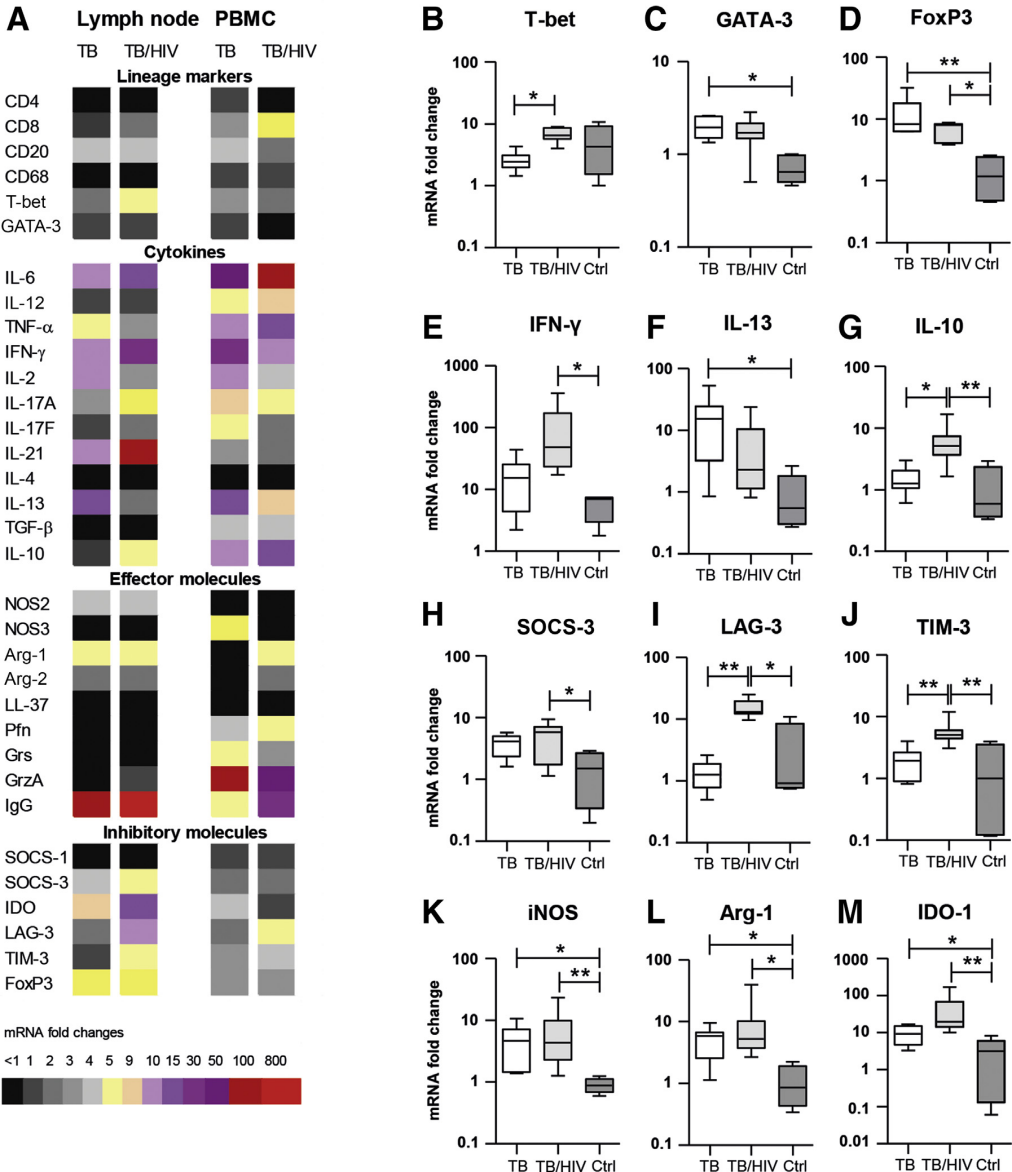
RNA profiling of lymph node tissues and peripheral blood mononuclear cells (PBMCs) from tuberculosis (TB)–infected and TB/HIV–co-infected patients. **A:** Heatmap demonstrating mRNA expression (fold change) of a number of immune mediators as determined by quantitative real-time PCR. **B**–**M:** mRNA expression of selected immune molecules in lymph node tissues. Data are expressed as medians ± ranges. *n* = 8 TB-infected patients (white bars); *n* = 8 TB/HIV–co-infected patients (light gray bars); *n* = 5 matched PBMC and lymph nodes tissue samples (dark gray bars). ∗*P* < 0.05, ∗∗*P* < 0.005 (Kruskal-Wallis and Dunn multiple comparisons tests). Arg, arginase; Ctrl, control; eNOS, endothelial nitric oxide synthase; FoxP3, forkhead box P3; GATA-3, GATA-binding protein 3; Grs, granulysin; GrzA, granzyme A; IDO-1, indoleamine 2,3-dioxygenase-1; IFN-γ, interferon-γ; iNOS, inducible nitric oxide synthase; LAG-3, lymphocyte activated gene-3; LL-37, human cationic antibacterial protein of 18 kDa; NOS, nitric oxide synthase; Pfn, perforin; SOCS, suppressor of cytokine signaling; TIM-3, T-cell immunoglobulin mucin-3; TNF-α, tumor necrosis factor-α.

Figure 4A illustrates mRNA expression profiling of lymph node tissue and peripheral blood cells from TB-infected compared with TB/HIV–co-infected patients. Overall, the data demonstrate that the transcriptional landscape at the site of TB infection may be relatively different compared with peripheral blood. Compared with HIV-negative TB-infected patients, PBMCs from TB/HIV–co-infected patients displayed a relative increase of CD8, IL-6, total IgG, Arg-1, and lymphocyte activated gene (LAG)-3 mRNA. In the lymph nodes, there was an increased mRNA expression of IL-6, IFN-γ, IL-21, Arg-1, total IgG, IDO, and forkhead box P3 (FoxP3) in both TB-infected and TB/HIV–co-infected tissues compared with the uninfected controls.

mRNA expression of selected molecules in TB-infected lymph nodes tissues compared with controls is demonstrated in Figure 4, B–M. TB/HIV–co-infected tissues expressed significantly more T-bet (*P* = 0.029), IFN-γ (*P* = 0.05), and IL-10 (*P* = 0.022) mRNA compared with TB (Figure 4, B, E and G), whereas the HIV-negative TB group displayed higher levels of Th2 markers, such as GATA-binding protein 3 (*P* = 0.016) and IL-13 (*P* = 0.014) mRNA (Figure 4, C and F). This finding is consistent with an up-regulation of the inflammatory chemokines CXCL9 and CXCL10 in TB/HIV co-infection (Supplemental Figure S1, B and C) and an enhanced tissue expression of the M2-marker CD163 in HIV-negative TB-infected patients (Figure 3, F and J). Moreover, FoxP3 expression was up-regulated in both TB-infected (*P* = 0.009) and TB/HIV–co-infected (*P* = 0.044) patients compared with the controls (Figure 4D). Likewise, the pleiotropic inhibitors suppressor of cytokine signaling (SOCS)-3, LAG-3, and T-cell immunoglobulin mucin (TIM)-3 were all up-regulated in TB/HIV co-infection compared with the controls (*P* = 0.05, *P* = 0.02, and *P* = 0.009, respectively), and LAG-3 and TIM-3 were also significantly higher in TB/HIV co-infection compared with TB infection (*P* = 0.005 and *P* = 0.009) (Figure 4, H–J).

**Supplemental Figure S3:**
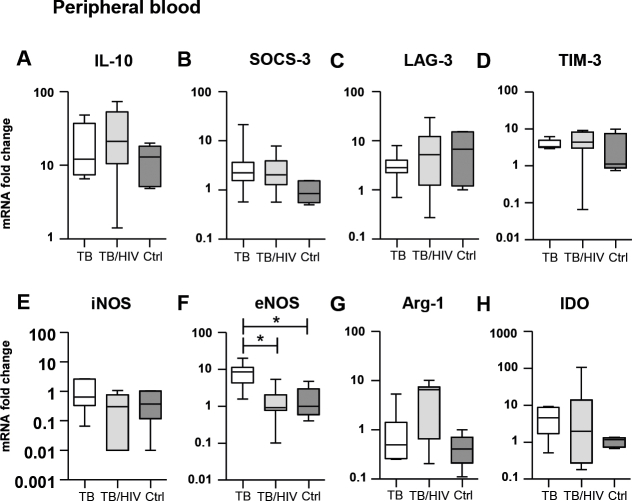
RNA profiling of peripheral blood mononuclear cells (PBMCs) from tuberculosis (TB)-infected and TB/HIV–co-infected patients using quantitative real-time PCR. **A**–**H:** mRNA expression of selected immune molecules in lymph node tissues. Data are expressed as medians ± ranges. *n* = 8 TB-infected patients (white bars); *n* = 8 TB/HIV–co-infected patients (light gray bars); *n* = 3 HIV-negative controls 4 HIV-positive controls (dark gray bars). ∗*P* < 0.05 (Kruskal-Wallis and Dunn multiple comparisons tests). Arg-1, arginase-1; Ctrl, controls; eNOS, endothelial nitric oxide synthase; IDO, indoleamine 2,3-dioxygenase; iNOS, inducible nitric oxide synthase; LAG-3, lymphocyte activated gene-3; SOCS-3, suppressor of cytokine signaling-3; TIM-3, T-cell immunoglobulin mucin-3.

Several T-cell–specific effector molecules as well as the innate antimicrobial peptide human cationic antibacterial protein of 18 kDa (LL-37) were expressed at low levels at the site of infection in TB and TB/HIV lymph node tissues (Figure 4A). Instead, the cell-signaling enzymes iNOS, Arg-1, and IDO were significantly up-regulated in both TB (*P* = 0.019, *P* = 0.036, and *P* = 0.019, respectively) and TB/HIV (*P* = 0.0072, *P* = 0.022, and *P* = 0.0012, respectively) compared with the control group (Figure 4, K–M). Importantly, differences in mRNA expression of most immune molecules shown in Figure 4 were only evident at the site of infection and not in corresponding samples from peripheral blood (Supplemental Figure S3).

### Differential Distribution of iNOS, Arg-1, and IDO Expression in *M. tuberculosis*–Infected Granulomatous Tissue

**Figure 5:**
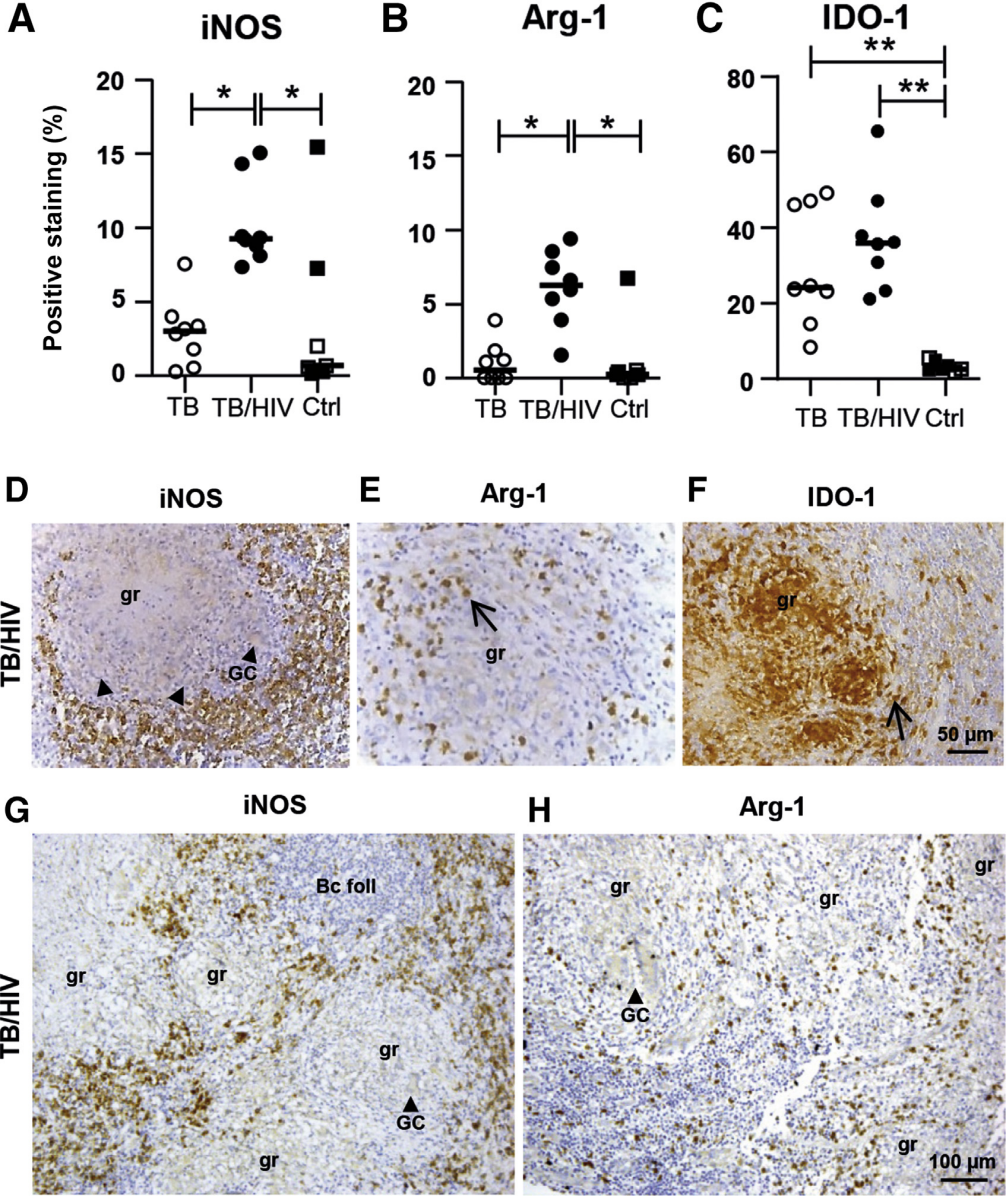
*In situ* expression of cell-signaling enzymes in tuberculosis (TB) infected–and TB/HIV–co-infected lymph node tissues. **A–C:** Immunohistochemistry and computerized image analyses were used to determine the expression (percentage of positive staining in the total cell area) and distribution of inducible nitric oxide synthase (iNOS) (**A**), arginase (Arg)-1 (**B**), and indoleamine 2,3-dioxygenase (IDO) (**C**). **D–F:** Representative images of iNOS (**D**), Arg-1 (**E**), and IDO (**F**) staining in TB/HIV–co-infected lymph node tissue. **G** and **H:** iNOS (**G**) and Arg-1 (**H**) at a lower magnification visualizing staining patterns in confluent granulomas. **Arrows** indicate positively stained cells, and **arrowheads** indicate giant cells (GCs). Data are expressed as medians ± interquartile ranges. *n* = 8 TB-infected patients (open circles); *n* = 8 TB/HIV–co-infected patients (closed circles); *n* = 3 HIV-negative controls (Ctrls) (open squares); *n* = 4 HIV-positive Ctrls (closed squares). ∗*P* < 0.05, ∗∗*P* < 0.005 (Kruskal-Wallis and Dunn multiple comparisons tests). Scale bars: 50 μm (**D–F**); 100 μm (**G** and **H**). Original magnification: ×16 (**D–F**); ×25 (**G** and **H**). Bc foll, B-cell follicle; gr, granuloma.

**Supplemental Figure S4:**
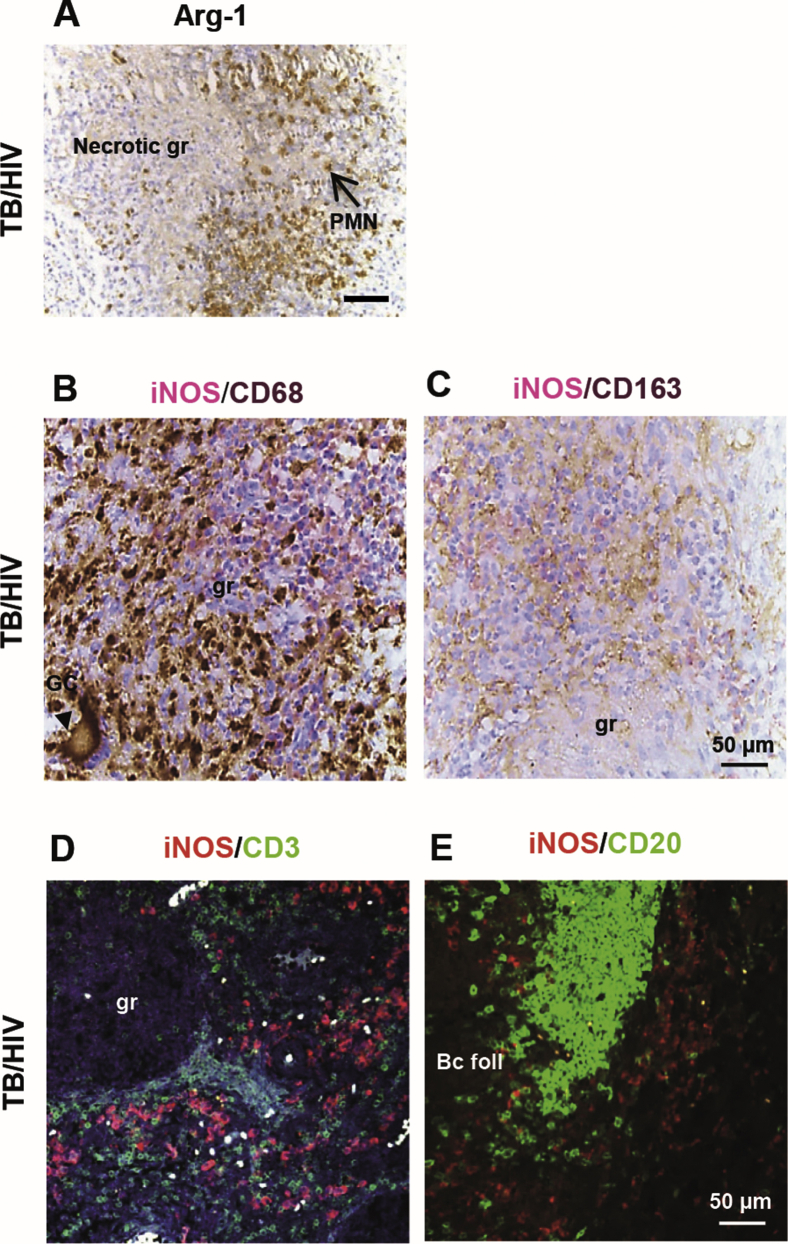
Multicolor microscopy was used to investigate co-expression of inducible nitric oxide synthase (iNOS) in different immune cells present in tuberculosis (TB)/HIV–co-infected lymph node tissue. **A:***In situ* expression of arginase (Arg)-1 visualized in the granulomatous tissue was particularly high in cells with a granulocytic morphology (**arrow**). **B–E:** iNOS (pink or red) expression was investigated using immunohistochemistry double staining with CD68 (brown) (**B**) or CD163 (brown) (**C**) or using immunofluorescence and confocal microscopy staining together withCD3 (green) (**D**) or CD20 (green) (**E**). For immunohistochemistry, iNOS was detected using a horseradish peroxidase–conjugated secondary antibody and double stained with diaminobenzidine. Representative images are shown in TB/HIV–co-infected lymph node tissue. Scale bars = 50 μm. Original magnification, ×25. Bc foll, B cell follicle; gr, granuloma; PMN, polymorphonuclear cell.

To further explore the phenotype and function of macrophages in the granulomatous tissue, the cell-signaling enzymes iNOS, Arg-1, and IDO were assessed. There was a significant increase of both iNOS (*P* = 0.003) and Arg-1 (*P* = 0.004) expression in the TB/HIV–co-infected group, although iNOS was also relatively higher in the TB-infected group compared with the controls (Figure 5, A and B). Notably, both iNOS (median of 4.64% HIV positive versus 0.29% HIV negative) and Arg-1 (median of 0.47% HIV positive versus 0.05% HIV negative) were up-regulated in the HIV-infected controls (Figure 5, A and B). Expression of the inhibitory molecule IDO was strongly enhanced (*P* = 0.0008) in both patient groups (Figure 5C). Interestingly, iNOS- and Arg-1–producing cells were mostly localized at the periphery of the TB granulomas in cells with a mononuclear morphology (Figure 5, D and E), whereas IDO-1 expression was detected primarily inside the granulomas (Figure 5F). Figure 5, G and H illustrates that iNOS and Arg-1 expression in and around confluent granulomas was mostly confined to the T-cell–rich areas surrounding the cores of the lesions. iNOS-expressing cells were also very few in the B-cell follicles (Figure 5G). Arg-1 expression was generally less stringent than iNOS, and numerous Arg-1–producing cells were found in the peripheral rim of the granulomatous lesions and in the lymphoid areas (Figure 5H). Multinucleated giant cells in the center of the granuloma expressed iNOS at a low intensity compared with the iNOS-positive cells surrounding the granuloma (Figure 5, D and G). Likewise, Arg-1 was expressed at lower intensity in giant cells and polymorphonuclear cells in and around the necrotic areas in the granulomas (Figure 5H and Supplemental Figure S4A).

### Elevated Expression of Lin^−^/CD33^+^CD15^+^/MAC387^+^/HLA-DR^int^/Arg-1^+^ Cells Characteristic of MDSCs in TB/HIV–Co-Infected Tissue

**Figure 6:**
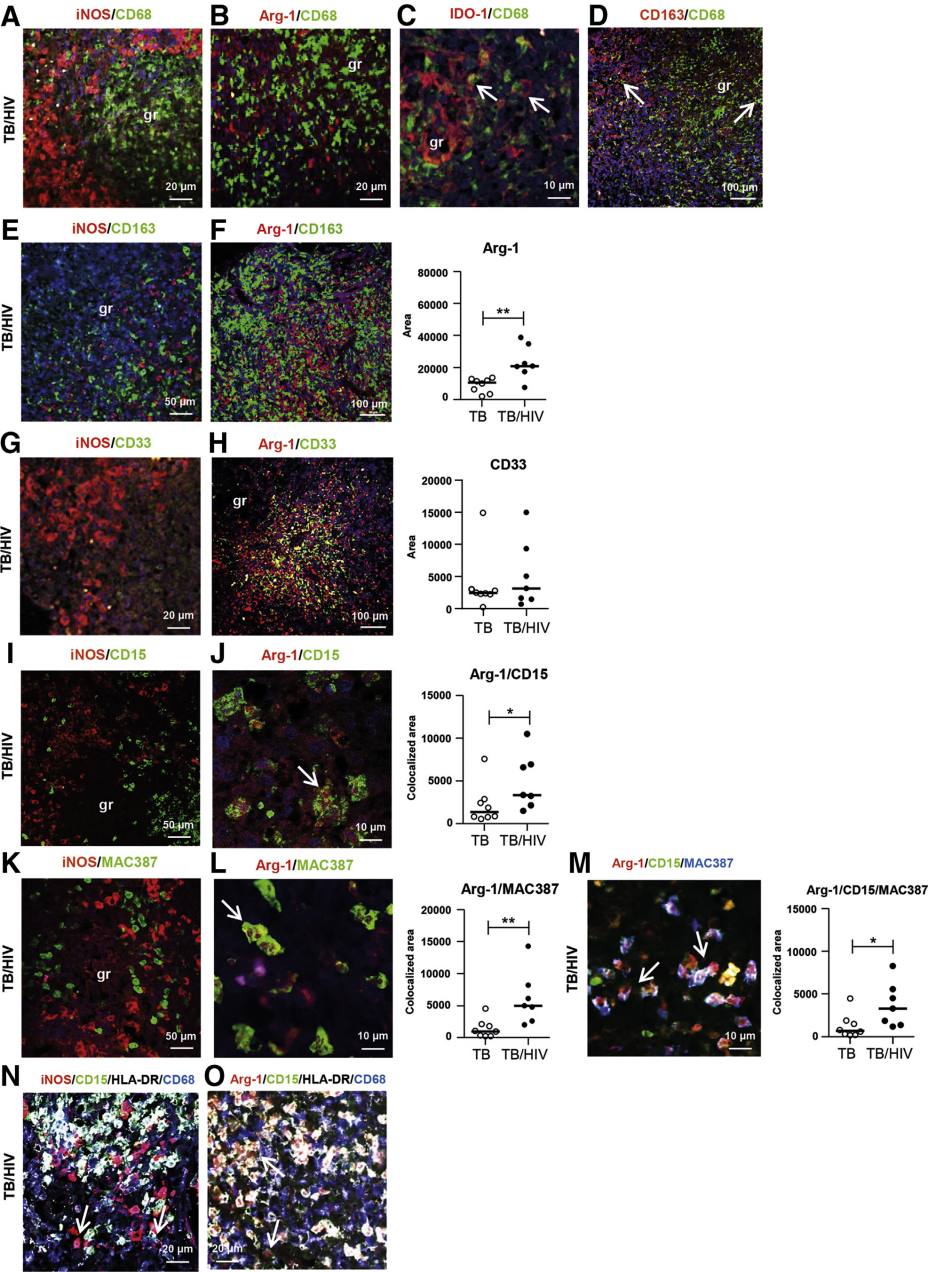
Confocal imaging and analyses of myeloid cell markers in tuberculosis (TB)–infected and TB/HIV–co-infected lymph node tissues. **A–O:** Immunofluorescence and confocal microscopy analyses were used to determine the expression (colocalized area) and distribution of the following markers: inducible nitric oxide synthase (iNOS) (red)/CD68 (green) (**A**), arginase (Arg)-1 (red)/CD68 (green) (**B**), indoleamine 2,3-dioxygenase (IDO) (red)/CD68 (green) (**C**), CD163/CD68 (green) (red) (**D**), iNOS (red)/CD163 (green) (**E**), Arg-1 (red)/CD163 (green) (**F**), iNOS (red)/(CD33) (green) (**G**), Arg-1 (red)/(CD33) (green) (**H**), iNOS (red)/(CD15) (green) (**I**), Arg-1 (red)/(CD15) (green) (**J**), iNOS (red)/(MAC387) (green) (**K**), Arg-1 (red)/(MAC387) (green) (**L**), Arg-1 (red)/(CD15) (green)/MAC387 (blue) (**M**), iNOS (red)/CD15 (green)/HLA-DR (white)/CD68 (blue) (**N**), and Arg-1 (red)/CD15 (green)/HLA-DR (white)/CD68 (blue) (**O**). Representative images of iNOS or Arg-1 double, triple, or quadruple stains are shown in TB/HIV**–**co-infected lymph node tissue. **F–M:** Positively stained area of Arg-1 (**F**) or CD33 (**H**) and colocalized area of Arg-1 and CD15 (**J**) or MAC387 (**L**) or Arg-1 and CD15 and MAC387 (**M**) was quantified using digital image analyses. **Arrows** indicate double-positive or triple-positive cells. Data are expressed as medians ± ranges. *n* = 8 TB-infected patients (open circles); *n* = 7 TB/HIV–co-infected patients (closed circles). ∗*P* < 0.05, ∗∗*P* < 0.005 (Mann-Whitney *U*-test). Original magnification: ×63 (**A, B, G, N**, and **O**); ×100 (**C, J, L**, and **M**); ×16 (**D, F**, and **H**); ×25 (**E, I**, and **K**). Scale bars: 20 μm (**A, B, G, N**, and **O**); 10 μm (**C, J, L**, and **M**); 100 μm (**D, F**, and **H**); 50 μm (**E, I**, and **K**). gr, granuloma.

Multicolour confocal microscopy demonstrated that neither iNOS nor Arg-1 co-localized with CD68-positive or CD163-positive macrophages (Figure 6, A, B, E and F). In contrast, IDO-1 expression co-localized largely with CD68^+^ macrophages, although IDO was also expressed in some CD68-negative cells (Figure 6C). Consistent with the nonoverlapping expression of CD68 and CD163 detected with IHC (Figure 3, I and J), IF confirmed that CD68 and CD163 co-expression was low in both TB-infected (data not shown) and TB/HIV–co-infected tissues (Figure 6D). Overall, co-expression of iNOS could not be detected in CD68^+^ or CD163^+^ cells, CD3^+^ T cells, or CD20^+^ B cells (Supplemental Figure S4, B–E) or in subsets, including CD56^+^ natural killers cells or CD123^+^ plasmacytoid DCs (data not shown), which were very few in the TB tissue. On testing of myeloid markers, iNOS did not co-localize with CD33^+^, CD15^+^, or MAC387^+^ (Figure 6, G, I and K) but was expressed in CD68-negative cells with low or intermediate HLA-DR expression (Figure 6N). Similar to iNOS, IDO was not expressed in CD15^+^ or MAC387^+^ cells (data not shown).

The confocal analyses further revealed that Arg-1–expressing cells were negative for lineage-specific markers CD3, CD20, CD56, and CD123 (data not shown). However, Arg-1 co-localized considerably with CD33^+^, CD15^+^, and MAC387^+^ cells with low or intermediate expression of HLA-DR (Figure 6, H, J, L, M, and O), suggestive of an MDSC cell subset. Quantification of co-expression revealed a significantly elevated expression of Arg-1^+^ (*P* = 0.006), Arg-1^+^MAC387^+^ (*P* = 0.002), Arg-1^+^CD15^+^ double-positive (*P* = 0.05), and Arg-1^+^CD15^+^MAC387^+^ triple-positive (*P* = 0.03) cells in TB/HIV co-infection compared with TB infection (Figure 6, F, J, L and M). TB/HIV–co-infected tissues demonstrated a high co-localization of Arg-1 with CD33^+^ cells located primarily around the core of the TB granulomas, although there was no difference in CD33 expression in TB-infected compared with TB/HIV–co-infected tissues (Figure 6H). Altogether, these results demonstrate the presence of elevated levels of cells characteristic of MDSCs in the microenvironment of lymph nodes granulomas from TB/HIV–co-infected patients.

### Elevated Levels of Granulocytic MDSCs in the Peripheral Circulation of TB/HIV–Co-Infected Patients

**Figure 7:**
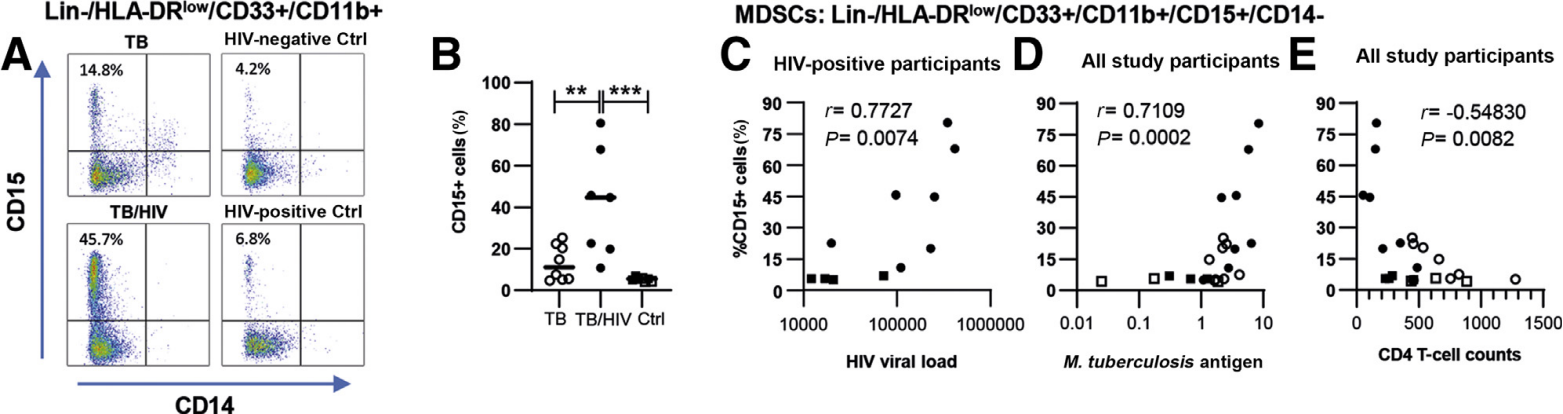
Flow cytometric analyses of cells characteristic of myeloid-derived suppressor cells (MDSCs) present in peripheral blood from tuberculosis (TB)–infected and TB/HIV–co-infected patients. **A:** Representative dot plots showing MDSCs present in TB-infected or TB/HIV–co-infected patients as well as HIV-negative and HIV-positive controls (Ctrls) based on gating of peripheral blood mononuclear cells (PBMCs) for Lin-negative, HLA-DR^low^, CD33^+^, and CD11b^+^ cells that were further divided into CD15^+^ and CD14^+^ cells. Frequency (percentage of CD15^+^ cells) of MDSCs in PBMC samples. **B**–**E:** CD15^+^ MDSCs in TB/HIV–co-infected and HIV-positive controls were positively correlated with plasma HIV viral load (**C**), whereas CD15^+^ MDSCs in TB-infected and TB/HIV–co-infected patients and Ctrls were positively correlated with *Mycobacterium tuberculosis* antigen load in lymph node tissue (**D**) but inversely correlated to peripheral CD4 T-cell counts (**E**) as determined using the Spearman correlation test. *n* = 8 TB-infected patients (open circles); *n* = 7 TB/HIV–co-infected patients (closed circles); *n* = 3 HIV-negative Ctrls (open squares); *n* = 4 HIV-positive Ctrls (closed squares). ∗∗*P* < 0.01, ∗∗∗*P* < 0.001 (Kruskal-Wallis and Dunn multiple comparisons tests). Ctrl, control.

**Supplemental Figure S5:**
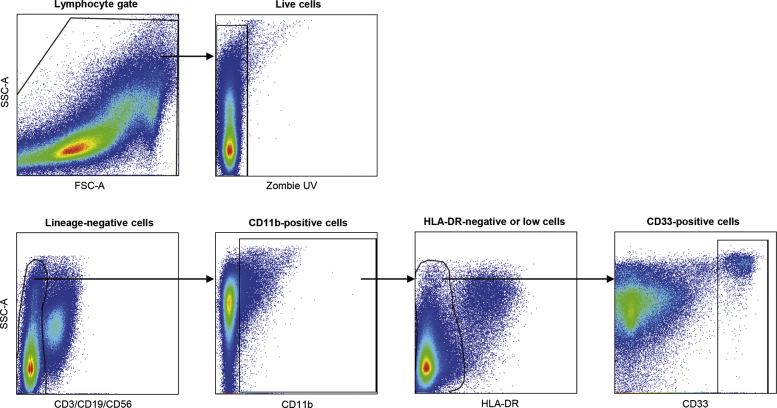
Gating strategy used to assess myeloid-derived suppressor cell–like cells present in peripheral blood from tuberculosis (TB)–infected and TB/HIV–co-infected patients. Representative dot plots from a TB/HIV–co-infected patient are shown. Live lymphocytes were gated for lineage-negative cells, and the consecutive gates for CD11b-positive cells, HLA-DR–negative or low cells, and CD33-positive cells are also indicated. The gates were set using unstained cell samples as well as fluorescence minus one controls. SSC, side scatter.

To explore whether MDSCs were present in the peripheral circulation of TB-infected patients, TB/HIV–co-infected patients, and controls, flow cytometry staining was performed using corresponding PBMC samples from the study participants. In humans, MDSCs have been characterized as monocytic CD11b^+^/CD14^+^ or granulocytic CD11b^+^/CD15^+^ MDSCs and are broadly defined as lymphocyte lineage-negative cells that express the common myeloid marker CD33 together with low or no expression of mature myeloid cell markers, such as HLA-DR, CD40, or CD80.^10^^,^^19^^,^^26^ Accordingly, a gating strategy was used for identification of monocytic (CD14^+^) or granulocytic (CD15^+^) MDSC subsets in peripheral blood based on lineage-negative (CD3, CD19, CD56) and HLA-DR–negative or low cells expressing the myeloid markers CD33^+^ and CD11b^+^ (Supplemental Figure S5).^27^ Flow cytometry showed that MDSCs identified among Lin^−^/HLA-DR^low^/CD33^+^/CD11b^+^ cells were mostly CD15^+^/CD14^−^, which is consistent with the phenotype of granulocytic MDSCs (Figure 7A). Indeed, CD15^+^ granulocytic MDSCs were significantly higher in TB/HIV–co-infected patients compared with TB-infected patients (*P* = 0.02) and controls (*P* = 0.0012) (Figure 7B). CD15^+^ MDSCs were also elevated in HIV-negative TB-infected patients, but this difference was not significant compared with the controls (Figure 7, A and B).

Correlation analyses further showed a positive correlation between the percentage of CD15^+^ MDSCs in peripheral blood and HIV viral load in plasma (HIV-positive participants; *r* = 0.77, *P* = 0.0074) or *M. tuberculosis* antigen load in tissue [HIV-positive participants; *r* = 0.82, *P* = 0.0033 (data not shown); all study participants; *r* = 0.71, *P* = 0.0002] but an inverse correlation between the percentage of CD15^+^ MDSCs and CD4^+^ T-cell counts in the peripheral circulation [HIV-positive participants; *r* = −0.70, *P* = 0.0204 (data not shown); all study participants; *r* = −0.55, *P* = 0.0082] (Figure 7, C–E). These results suggested that MDSCs are prevalent in TB/HIV–co-infected patients, both at the site of *M. tuberculosis* infection and in the systemic circulation, and that MDCSs may be associated with progression of TB disease in HIV-positive individuals.

## Discussion

This study demonstrates enhanced chronic inflammation with an accompanying immunosuppressive microenvironment in lymph node granulomas from TB/HIV–co-infected compared with HIV-negative TB-infected patients. TB/HIV co-infection was characterized by poorly formed, necrotic granulomas that displayed a particularly high *M. tuberculosis* antigen expression. Persistent immune activation in TB/HIV co-infection involved not only enhanced production of proinflammatory (IL-6, IFN-γ, CXCL9, CXCL10, and iNOS) as well as anti-inflammatory (IL-1RA, IL-10, Arg-1, and IDO) mediators but also elevated numbers of myeloid cells with an aberrant phenotype and morphology characteristic of MDSCs in TB-infected lymph nodes. Elevated IgG responses in TB, and particularly in TB/HIV–co-infected patients, are indicative of chronic immune activation and active TB disease progression. On the other hand, reduced T-cell numbers and low mRNA expression of antimicrobial and cytolytic effector molecules, such as perforin and granulysin, were evident in both HIV-negative TB-infected and TB/HIV–co-infected tissues, whereas immunoregulatory FoxP3 and IDO expression was enhanced. Furthermore, Arg-1 was higher in MDSC-like cells in TB/HIV–co-infected tissues, and granulocytic MDSCs were significantly increased in peripheral blood from TB/HIV–co-infected compared with HIV-negative TB-infected patients. MDSC counts correlated positively with HIV viral load in plasma as well as *M. tuberculosis* antigen load in tissue but were inversely correlated with peripheral CD4 T-cell counts, which may indicate a role for MDSCs in TB/HIV disease progression.

Pathological features of chronic inflammatory diseases and solid cancers typically include enhanced levels of proinflammatory mediators, such as cytokines, chemokines, and reactive oxygen and nitrogen species. Emerging evidence supports the concept that chronic inflammation promotes local immunosuppression via induction of proinflammatory mediators and the accumulation and activation of immunosuppressive cells.^28^ In line with that view, differentiated macrophages also produce anti-inflammatory cytokines, most notably IL1-RA and IL-10, which are up-regulated by proinflammatory mediators and engage in autocrine signaling.^29^ IL-10 reduces HLA-DR expression and antigen presentation in macrophages and inhibits the production of proinflammatory cytokines.^30^ Likewise, MDSCs expand during various pathological conditions associated with acute or chronic inflammation.^19^ This paradox may represent both immune exhaustion and active immune inhibition. In TB, defective activation of myeloid cells^8^^,^^13^ may contribute to impaired TB immunity by suppression of Th1 effector cells and activation of different pathogenic and Treg cell subsets with a large phenotypic and functional heterogeneity, including expression of immune checkpoint molecules, such as IDO, cytotoxic T-lymphocyte-associated protein (CTLA)-4, programmed death ligand (PD-L)1, and LAG-3.^14^ Although excessive inflammation in TB is harmful and an anti-inflammatory response is required to avoid tissue destruction, local polarization of immunosuppressive subsets with poor antibacterial properties may also delay or prevent the clearance of infection, leading to progressive TB disease. We previously found that high iNOS expression in TB granulomas coincides with impaired Th1 and CD8^+^ CTL responses but enhance FoxP3^+^ Treg cells in patients with active TB.^21^ The antimicrobial peptides LL-37 and granulysin were reduced in TB lesions from human lung^22^^,^^23^ and lymph nodes,^21^ whereas Th2 (IL-4, IL-13, and CCL4) and Treg (FoxP3, CTLA-4, glucocorticoid-induced tumor necrosis factor receptor–related protein, and transforming growth factor-β) responses were enhanced together with excess expression of suppressors of cytokine signaling (SOCS-1 and -3).^21^^,^^25^ In this study, the localization of Arg-1^+^ MDSC-like cells outside the core of the granuloma in close contact with CD3^+^ T cells may partly explain the mixed immune response profile in TB/HIV–co-infected lymph nodes, including induction of both inflammatory and immunosuppressive or anti-inflammatory mediators as determined using mRNA expression analyses.

TB granulomas share many similarities with the microenvironment in solid tumors, including regions of hypoxia and necrosis, extensive fibrosis, and local immunosuppression. Immunosuppressive cell subsets, such as MDSCs, Treg cells, and T cells, expressing immune checkpoint inhibitors are known to play important roles in tumor immune evasion. MDSCs located within tumor tissue are typically highly immunosuppressive.^19^^,^^31^ In particular, both circulating and tumor-infiltrating MDSCs typically express high levels of Arg-1,^32^^,^^33^ and high arginase activity at the tumor site correlates with decreased cytokine production and low levels of the T-cell receptor (TCR) CD3 ζ-chain.^34^ In patients with non–small cell lung cancer, granulocytic MDCSs in peripheral blood were found to be inversely correlated with CD8^+^ T-cell frequencies.^35^ In those with renal or lung carcinoma, either depletion of MDSCs or inhibition of Arg-1 was found to reestablish T-cell proliferation and TCR CD3 ζ-chain expression.^34^^,^^36^ Recent studies also demonstrated that MDSCs mainly exert immunosuppressive functions through the induction of Arg-1 and L-arginine depletion.^37^, ^38^, ^39^ Circulating MDSCs in patients with gastric cancer expressed Arg-1 that was associated with decreased CD8^+^ T-cell proliferation as well as with reduced IFN-γ production and granzyme B secretion from those cells.^40^ Similarly, elevated levels of MDCSs in patients with chronic hepatitis C infection are associated with a down-regulation of the TCR ζ-chain on CD8^+^ T cells.^41^ MDSCs in this inflammatory environment produce Arg-1 and increased Arg-1^+^ cells in the liver were closely associated with tissue pathology in patients with chronic hepatitis.^41^ Recently, it was also reported that monocytic MDSCs expanded in blood from patients with coronavirus disease 2019 and were strongly associated with disease severity, including suppressed T-cell proliferation and IFN-γ production as well as down-regulated expression of the CD3 ζ-chain, partly via an Arg-1–dependent mechanism.^42^

Despite extensive evidence of MDSCs in different types of cancer, relatively few studies have investigated these cells in either HIV or TB infection, and assessments of MDSCs at the site of infection are scarce. In HIV infection, the original findings suggest that peripheral granulocytic MDSCs were increased in highly active antiretroviral therapy–naive HIV-positive patients but rapidly declined after the start of therapy.^9^ Similar to the results of this study, MDSC numbers in blood showed a positive correlation with HIV viral loads and a negative correlation with CD4^+^ T-cell counts.^9^ Enhanced levels of immunosuppressive monocytic MDSC have also been found in HIV-infected patients despite 2 years of effective antiretroviral therapy.^43^ Uncontrolled HIV replication has been observed to result in persistent immune activation, including elevated IL-6 levels, that mediates an expansion of MDSC numbers in HIV-infected patients.^44^^,^^45^ Furthermore, HIV-induced MDSCs may increase the expression of inhibitory mediators, including IL-10 and PD-L1 protein,^46^ restrict proliferation of effector T cells, and instead promote the differentiation of FoxP3^+^ Treg cells in T-cell co-cultures.^9^^,^^44^^,^^46^ Accordingly, granulocytic MDSCs expressing PD-L1 protein could inhibit both proliferation and IFN-γ production of CD8^+^ T cells from HIV-infected patients.^47^ Another report observed an inverse correlation between granulocytic MDSCs and expression of the TCR CD3 ζ-chain in HIV-infected patients that was restored on *in vitro* depletion of MDSCs.^48^

In human TB, MDCSs that suppress T-cell functions were initially described in the peripheral blood and in pleural fluid from patients with active TB.^8^ MDSC levels in patients with active TB were comparable to those in patients with cancer and contributed to reduced Th1 cytokine expression, whereas proinflammatory cytokines, such as IL-1β, IL-6, IL-8, and monocyte chemoattractant protein-1, were increased.^8^ High frequencies of granulocytic MDSCs have been observed in both peripheral blood and bronchoalveolar lavage specimens from patients with active pulmonary TB that suppressed T-cell proliferation, whereas MDSC were rapidly reduced on effective anti-TB treatment.^49^ Increased MDSC levels in peripheral blood from TB-infected children have also been linked to heightened IDO and Arg-1 levels in matched plasma samples.^50^ Furthermore, *in vitro* generated MDSCs promote *M. tuberculosis* replication by altering granuloma structure and stability and shift the balance of soluble mediators at the site of infection toward suppressive and regulatory cytokines, including a high release of IL-10 and IL-6,^51^ which is consistent with the mRNA profiling data in the current study. MDSCs from HIV-infected individuals have also demonstrated defective innate immunity to *M. tuberculosis* and exhibited a higher intracellular replication of *M. tuberculosis* compared with HLA-DR^hi^ myeloid subsets.^52^ This finding is consistent with an enhanced *M. tuberculosis* antigen expression in TB/HIV–co-infected lymph nodes as well as elevated *M. tuberculosis* antigen load in TB/HIV granulomas compared with HIV-negative TB, which may imply that HIV contributes to an immune cell dysfunction locally in the tissue that will make macrophage subsets more permissive to intracellular growth of *M. tuberculosis*. In turn, an enhanced bacterial replication may foster the expansion of MDSCs in TB/HIV–co-infected patients.

In TB-HIV–co-infected lymph node granulomas, IDO was mostly expressed in CD68^+^ macrophages, which is consistent with previous findings.^53^ In contrast, iNOS was abundantly expressed around the CD68^+^ core of the granulomas, although we were not able to fully characterize the phenotype of iNOS-producing cells. Immunosuppressive MDSCs from patients with pulmonary TB correlated with depleted serum levels of L-arginine but increased levels of NO, suggesting that MDSCs may exert their suppression by enhanced iNOS activity.^49^ In line with this finding, BCG vaccination of mice resulted in infiltration of NO-producing MDSCs into the skin that were unable to kill BCG bacteria but instead impaired T-cell priming in the draining lymph nodes.^54^ iNOS-expressing cells with immunosuppressive functions resembling monocytic MDSCs have also been identified in HIV and simian immunodeficiency virus infection.^55^ Because NO production by myeloid cells can contribute to suppressive T-cell responses, it is possible that iNOS-expressing cells observed in TB/HIV–co-infected lymph node tissue also represent a subset of suppressive myeloid cells.

Recent evidence suggests that pharmacologic exhaustion of MDSCs with the small-molecule inhibitor tasquinimod resulted in reduced FoxP3^+^ Treg cells but enhanced CD8^+^ effector T cells in the lung of *M. tuberculosis*–infected mice, and tasquinimod-mediated depletion of MDSCs also enhanced *M. tuberculosis* clearance in the lungs.^56^ In addition, tasquinimod impaired the formation of organized TB granulomas that contain cores of neutrophils with MDSC-like properties.^57^ In the microenvironment of solid tumors, tasquinimod treatment was also observed to decrease Arg-1 and CD206 mRNA expression in macrophages, indicating a shift from M2-like myeloid cells to the M1 phenotype.^58^ In a mouse lung tumor model, inhibition of Arg-1 in myeloid cells diminished growth of established tumors by increasing T-cell infiltration into the tumor tissue, which was accompanied by a significant increase in the CD8/Foxp3 ratio and IFN-γ production in T cells isolated from the tumors.^59^ Similarly, another small-molecule inhibitor of Arg-1 reversed myeloid cell–mediated immunosuppression and restored T-cell proliferation in different solid tumors, supporting the value of Arg-1 as an immunomodulatory target.^60^ These reports suggest that inactivation or depletion of MDSC subsets or a shift toward proinflammatory myeloid cells might have potential in effective host-directed therapy that could be a powerful adjunct to conventional antibiotic therapy in chronic TB and TB-HIV co-infection.
